# A novel three-part pharynx and its parallel evolution within symbiotic marine nematodes (Desmodoroidea, Stilbonematinae)

**DOI:** 10.1007/s13127-024-00643-0

**Published:** 2024-07-05

**Authors:** Philipp Pröts, Veronica Novotny-Diermayr, Jörg A. Ott

**Affiliations:** 1 Department of Functional and Evolutionary Ecology, University of Vienna, Vienna, Austria; 2 Experimental Drug Development Centre (EDDC), A*Star, Singapore, Singapore

**Keywords:** Symbiosis, Nuclei, Musculature, Homology, Glands, Function

## Abstract

Stilbonematinae are nematodes commonly found in shallow marine sands. They are overgrown by a genus- and species-specific coat of chemoautotrophic sulphur-oxidizing ectosymbiotic bacteria which profit from the vertical migration of their hosts through the chemocline by alternately gaining access to oxidizing and reducing chemical species, while in return, the host feeds on its symbionts. The subfamily exhibits a large morphological variability; e.g. the anterior pharynx is cylindrical in genera possessing a voluminous coat, but species with a bacterial monolayer possess a distinctly swollen corpus and therefore a tripartite pharynx. Since 18S-based phylogenetic analyses do not show close relationships between corpus-bearing species, we investigated the pharynx morphology using phalloidin staining in combination with confocal laser scanning microscopy, transmission electron microscopy and light microscopy in order to assess an independent evolution. The class-wide stable position of the subventral pharynx ampullae was used as a morphological marker. Ampullae are positioned at the anterior-most end of the isthmus in *Cyathorobbea*, further posterior in *Catanema* and *Robbea* and inside the corpus in *Laxus oneistus*. We therefore conclude an independent evolution of corpus enlargements within Stilbonematinae. This further suggests that pharynx morphology is driven by the volume of the symbiotic bacterial coat rather than phylogeny. Based on an existing mathematical model, an enlarged corpus should enable its bearer to ingest food in smaller quantities, in gourmet style, whereas a cylindrical pharynx would restrict its bearer to ancestral gourmand feeding. A review of pharynx types of Nematoda showed that the Stilbonematinae pharynx is substantially different compared to other tripartite pharynges. The lack of pharyngeal tubes and valves, the undivided corpus and evenly distributed nuclei in the isthmus warrant the definition of the “stilbonematoid” three-part pharynx.

## Introduction

Stilbonematinae is a subfamily of free-living marine nematodes remarkable for their symbiosis with bacterial ectosymbionts (Polz et al., 1992). These symbionts belong to the Gammaproteobacteria genus *Candidatus* Thiosymbion Zimmermann et al., 2016 and cover most of their host’s body surfaces (Ott et al., 2004). The category “Candidatus” serves as a prefix in bacterial taxonomy to indicate so far uncultivable bacterial clades (Murray & Schleifer, 1994; Oren, 2021). The symbiosis is highly specific in that each host species possesses a unique and single morpho- and phylotype of bacteria (Zimmermann et al., 2016). Although the worm genera in this subfamily are phylogenetically closely related, they show extraordinary differences in body length and shape, the structure of the cuticle and cephalic capsule, the shape of the external opening of the lateral sense organs (*fovea amphidialis*) and the construction of the pharynx.

Such a remarkable morphological repertoire may be more expected for a higher systematic unit such as an order than for a subfamily. Likewise, the symbiotic bacteria differ in cell shape and the geometric arrangement of the coat they form (Scharhauser et al., 2020). This may be a monolayer of rods or coccobacilli as is found in genera such as *Catanema* Cobb, 1920; *Robbea* Gerlach, 1956; *Laxus* Cobb, 1894 and *Cyathorobbea* Scharhauser et al., 2024. For genera such as *Eubostrichus* Greef, 1869 and *Eubostrichopsis* Ott & Pröts, 2021, a complex monolayer with elongated filamentous bacteria is characteristic (Ott & Pröts, 2021; Ott et al., 2014). In all the above cases, each bacterium is in direct contact with the host’s cuticle. The third configuration of bacterial coating involves a multilayer of cocci, as in the genera *Paralaxus* Scharhauser et al., 2020, *Leptonemella* Cobb, 1920 and *Stilbonema* Cobb, 1920.

*Candidatus* Thiosymbion is a sulphur-oxidizing chemoautotroph (Polz et al., 1992; Zimmermann et al., 2016). By moving through the chemocline between upper oxidized and lower reduced layers, the host provides its symbionts with the required chemical species for sulphur oxidation, in particular oxygen and sulphide, respectively (Ott et al., 1991). Gut content observations and stable isotope (^13^C) composition are strong evidence that the host in return gains all or most of its nutrition by grazing on its own bacterial coat (Ott et al., 1991).

The nematode pharynx is the main organ for food acquisition. It is extremely diverse in both form and function and is of great phylogenetic significance (Bird & Bird, 1991; Chitwood & Chitwood, 1977; Maggenti, 1981). Its variability within the phylum is the product of numerous cases of both convergent and divergent evolution (Chitwood & Chitwood, 1977). Despite its variability, however, morphological commonalities can be identified. The basic nematode pharynx consists of several tissue types: muscle, gland, nerve and epithelial tissue, which reflects its ectodermal origin (Bird & Bird, 1991). It is characterized by a triradiate cuticle-lined lumen and is surrounded by a basal extracellular matrix (ECM). In cross section one ray of the lumen points towards the ventral side while the other two are oriented in dorsolateral direction. Therefore, one dorsal and two subventral sectors can be identified (Chitwood & Chitwood, 1977; Decraemer et al., 2014). Each sector consists of two neighbouring radial muscles that are one sarcomere long and extend from the cuticle to the basal ECM (Decraemer et al., 2014; Roggen, 1970). Two neighbouring sectors are delimited from each other by a marginal cell that surrounds each cuticular apex of the triradiate lumen. Between the sarcomeres in each sector, gland ducts extend through the pharynx. They originate from either three (one in each sector) or five (one dorsal and four ventrolateral) uninuclear gland cells at the posterior end of the pharynx and open into the pharynx lumen at specific positions (Chitwood & Chitwood, 1977; Decraemer et al., 2014). Within this basic configuration, three pharynx types can be distinguished: one-part, two-part and three-part pharynges (Allen, 1960; Bird & Bird, 1991; Maggenti, 1981). The one-part pharynx is considered ancestral and is a simple cylindrical muscular tube without apparent subdivisions. The two-part pharynx is characterized by a cylindrical anterior part and wider posterior bulbus while the three-part pharynx is further subdivided into a corpus, a narrow isthmus and a posterior bulbus (Allen, 1960; Bird & Bird, 1991; Maggenti, 1981).

A common morphological trait in Chromadorida and Desmodorida (Stilbonematinae belong to the latter) is a simple two-part pharynx with a more or less pronounced posterior bulbus (Holovachov, 2019; Tchesunov, 2014a). These ancestral chromadorean pharynx proportions are found in the stilbonematine genera *Eubostrichus*, *Eubostrichopsis*, *Leptonemella*, *Stilbonema* and *Paralaxus.* In other genera, such as *Catanema*, *Robbea*, *Cyathorobbea* and the species *Laxus oneistus* Ott et al., 1995, however, a distinct muscular corpus swelling is apparent. Our hypothesis is that feeding on their symbionts may provide an explanation for the variability of the pharynx of Stilbonematinae. From this perspective, the diversity in the morphology of the pharynx as the organ for food acquisition would reflect the diversity of the genus- and species-specific arrangement of the symbionts on their host. Accordingly, in relatively high-volume bacterial coats (i.e. a complex monolayer or a multilayered coat), the host pharynx has a cylindrical anterior part, whereas species with a bacterial monolayer mostly exhibit a distinctly swollen corpus (Scharhauser et al., 2020).

Recent phylogenies based on both 18S rRNA and COI sequences show no close relationships between those taxa having a muscular, swollen corpus (Leduc & Sinniger, 2018; Scharhauser et al., 2020). Here, we show that the corpus swellings evolved several times independently within this subfamily. This result is based on state-of-the-art methods such as phalloidin staining in combination with confocal laser scanning microscopy, ultra-thin sectioning in combination with transmission electron microscopy, light microscopy and computational 3D visualization. We argue that the geometry (i.e. high volume vs. low volume) of the symbiotic coat was a decisive evolutionary force in the development of a specialized pharynx type.

Other three-part pharynx types are known for orders within Nematoda which are not closely related to Desmodorida. Such types can be found within Plectida, Rhabditida, Diplogasteromorpha and Tylenchomorpha. In order to demarcate the Stilbonematinae three-part pharynx from already known tripartite pharynx types, we review the pharynx types defined so far for Nematoda and introduce a novel type of three-part pharynx, the “stilbonematoid” pharynx.

## Materials and methods

### Collection and fixation

Sediment containing individuals of *Cyathorobbea hypermnestra*, *Cy. agricola*, *Cy. ruetzleri* Scharhauser et al., 2024, *Paralaxus cocos* Scharhauser et al., 2020, *Laxus oneistus* Ott et al., 1995 and *Robbea judithae* Scharhauser et al., 2024 was collected in February 2019 by hand from subtidal marine sand near Carrie Bow Cay, Belize. Specimens of *Catanema schiemeri* Ott et al., 2020, *Robbea lotti* and *R*. *weberae* Scharhauser et al., 2024 were extracted from sand collected in the bay of Sant Andrea on Elba, Italy, in September 2020. Specimens of *Eubostrichus topiarius* Berger et al., 1996 and further specimens of *Catanema schiemeri* were collected in Vestar, Rovinj, Croatia in 2018. For autofluorescence imaging, specimens of *Catanema* sp. were collected in Guadeloupe in 2016. For immunohistochemistry, samples were relaxed in a MgCl_2_ solution isosmotic to seawater, anterior ends were separated with a razor blade and subsequently fixed in 4% paraformaldehyde in 1 × phosphate-buffered saline (PBS, pH 7.3) for 1 h at room temperature, rinsed afterwards in 1 × PBS three times for 15 min each and stored in 1 × PBS until further processing.

### Transmission electron microscopy

Samples of *Laxus oneistus* from the collection of JAO were originally fixed and embedded as described in Nebelsick et al. (1992). Ultra-thin sections were produced using a Leica UC6 microtome (Leica Microsystems, Wetzlar, Germany) and transferred to formvar-coated copper slot grids for subsequent contrasting with 1% aquatic uranyl acetate for 20 min, rinsed in _dd_H_2_O, treated with lead citrate for 5 min and again rinsed in _dd_H_2_O. Analysis and image acquisition were performed on a iTEM Zeiss Libra 120 electron microscope (Carl Zeiss, Oberkochen, Germany) equipped with the software iTEM.

Additional TEM negatives of *Cyathorobbea hypermnestra* and *Catanema schiemeri* (collected in Carrie Bow Cay, Belize and Vestar, Rovinj, Croatia, respectively) from the collection of JAO were converted into positives and used for this paper.

Samples of *Cyathorobbea hypermnestra* were fixed in 4% glutaraldehyde and 2% osmium tetroxide (OsO_4_) in 0.2 M sodium cacodylate buffer (pH 7.2), rinsed in the same buffer and postfixed in 2% OsO_4_ in 0.2 M sodium cacodylate buffer. Fixation and first hydration steps were done on ice, whereas subsequent dehydration steps were done at room temperature. Dehydrated specimens were infiltrated in a series of pure ethanol to Spurr resin mixtures of the following ratios: 2:1, 1:1, 1:2, respectively, before embedding in pure Spurr epoxy resin (Spurr, 1969) and polymerizing at 70 °C for 12 h.

Samples of *Catanema schiemeri* were fixed after Eisenman and Alfert (1982), dehydrated in ethanol and embedded in Spurr epoxy resin.

### Immunohistochemistry, autofluorescence and confocal microscopy

#### Phalloidin and nuclei staining

Specimens were transferred into a solution of 0.1 M phosphate buffer (pH 7.3), 2.5% Triton-X and 2% DMSO for permeabilization overnight. Afterwards, they were treated with Alexa Fluor 488 phalloidin (dilution 1:50, Molecular Probes, Eugene, OR, USA) and DAPI (dilution 1:120, Invitrogen, Carlsbad, CA, USA) in phosphate buffer overnight to label F-actin filaments and nuclei, respectively. Samples were then rinsed three times for 30 min each in 1 × PBS and mounted on standard microscope slides with Fluoromount G (Southern Biotech, Birmingham, AL, USA). The slides were kept at 4 °C prior to examination.

#### Autofluorescence

Animals were fixed in 4% PFA for 1 h and transferred to glycerol to water 1:9, allowing the mixture to slowly evaporate over several days before mounting in pure glycerol on microscope slides.

Images were acquired on a Leica SP5 II confocal laser scanning microscope (Leica Microsystems, Wetzlar, Germany). Image stacks were created using the LAS AF Software. Image processing such as background reduction or contrast enhancement was performed using the open-source software Fiji (RRID:SCR_002285; Schindelin et al., 2012) with the implemented CLAHE module (Zuiderveld, 1994); the open-source scientific visualization software Drishti (RRID:SCR_017999; Limaye, 2012) and Amira 6.4 (RRID:SCR_007353) were used for 3D volume rendering. Inkscape (RRID:SCR_014479) was used for schematic drawings and GIMP (RRID:SCR_003182; www.gimp.org) to remove small dust particles from light microscopy images.

## Results

### Gross morphology of the stilbonematoid pharynx

**Fig. 1:**
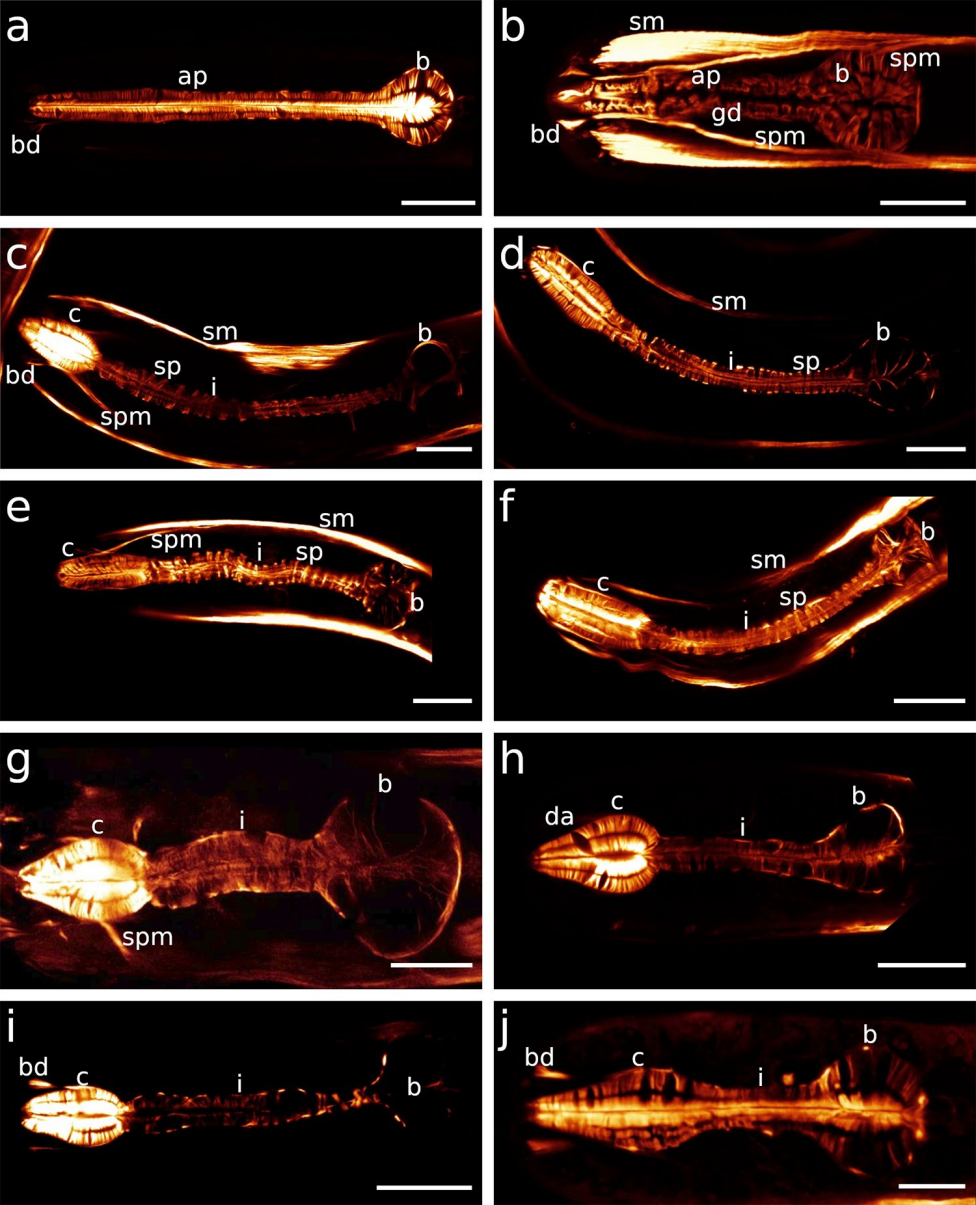
Pharynx musculature of Stilbonematinae, CLSM optical sections. Anterior on the left side. **a** *Eubostrichus topiarius*, **b** *Paralaxus cocos*, **c** *Catanema schiemeri*, **d** *Robbea lotti*, **e** *Robbea weberae*, **f** *Robbea judithae*, **g** *Cyathorobbea hypermnestra*, **h** *Cyathorobbea ruetzleri*, **i** *Cyathorobbea agricola*, **j** *Laxus oneistus*. Scalebars 20 μm. ap, anterior pharynx; b, bulbus; bd, buccal dilator muscle; c, corpus; da, dorsal ampulla; gd, gland duct; i, isthmus; sp, spiral muscle; spm, somato-pharyngeal muscle; sm, somatic muscle

**Fig. 2:**
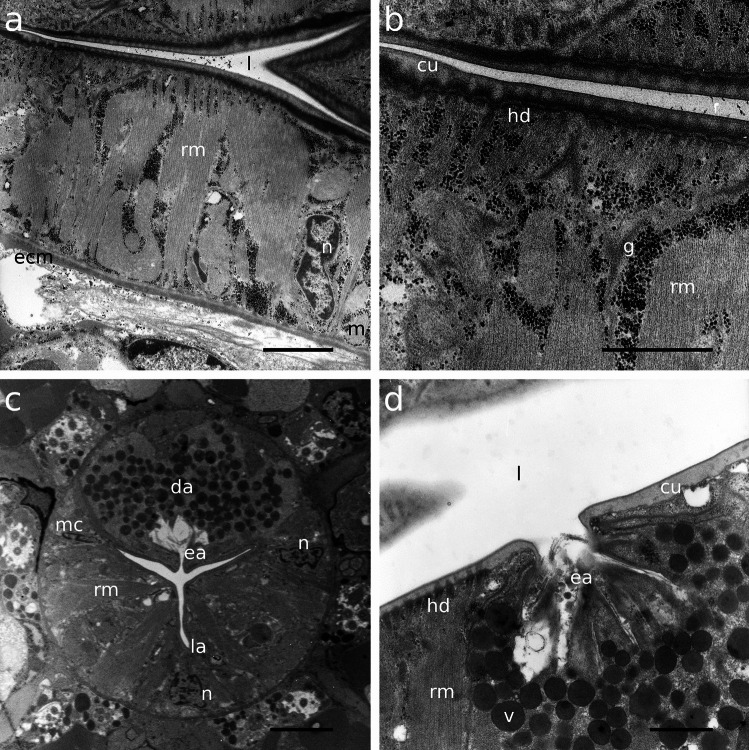
Transmission electron micrographs of the corpus musculature and gland ampullae. **a**, **b** *Catanema schiemeri*, **c**, **d** *Cyathorobbea hypermnestra*. Scalebars **a** 2 μm; **b** 1 μm; **c** 2 μm; **d** 1 μm. cu, cuticle; da, dorsal ampulla; ea, end apparatus; ecm, extracellulary matrix; g, glycogen; hd, hemidesmosomes; l, lumen; la, lumen apex; m, mitochondrium; mc, marginal cell; n, nucleus; rm, radial muscle; v, vesicle

**Fig. 3:**
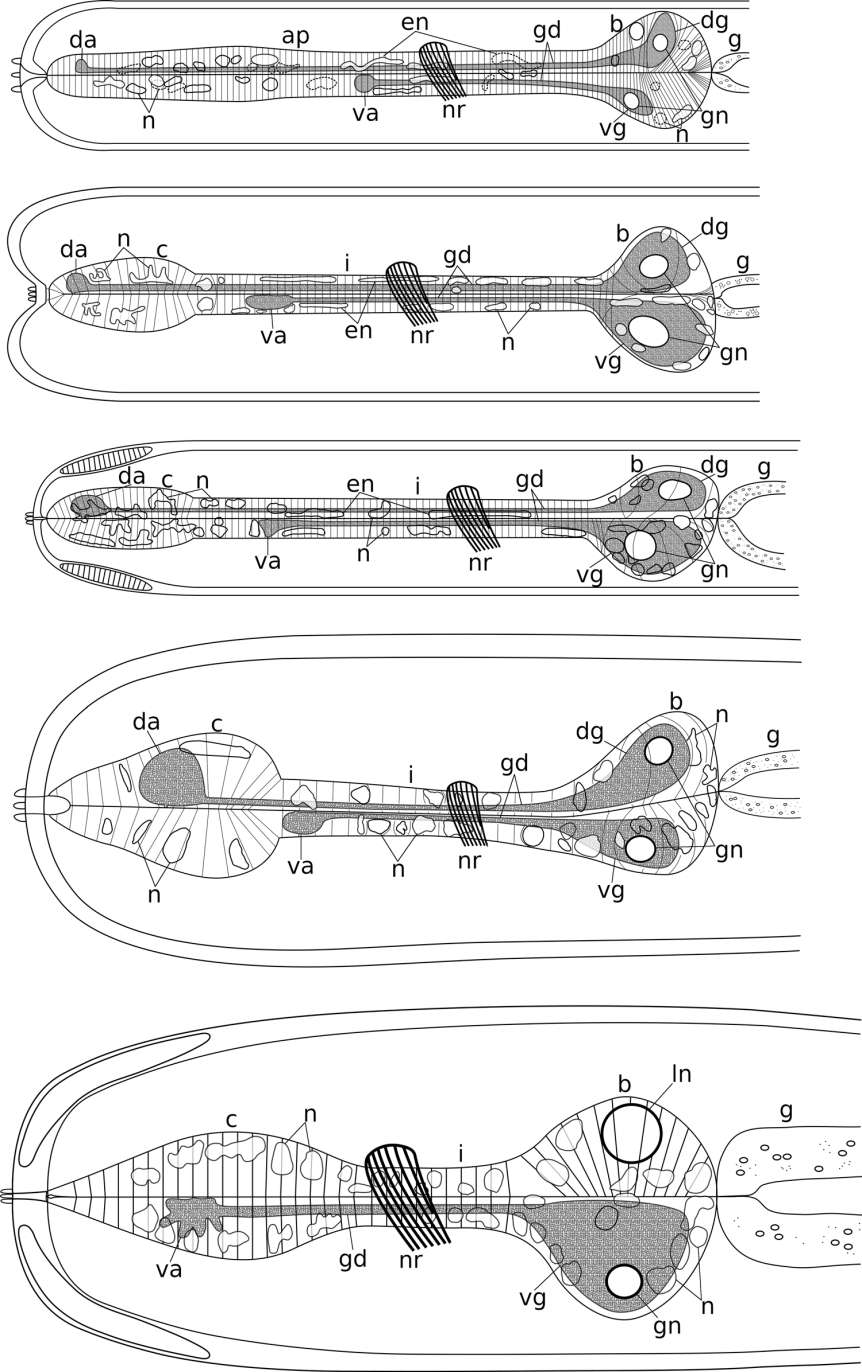
Morphological diversity of the stilbonematoid pharynx. From top to bottom: *Eubostrichus topiarius*, *Catanema schiemeri*, *Robbea lotti*, *Cyathorobbea ruetzleri*, *Laxus oneistus*. Pharynx lengths are aligned. Abbreviations: ap, anterior pharynx; b, bulbus; c, corpus; da, dorsal ampulla; dg, dorsal gland; en, elongated nucleus; g, gut; gd, gland duct; gn, gland nucleus; i, isthmus; ln, large nucleus; n, nucleus; nr, peripharyngeal nerve ring; va, subventral ampulla; vg, subventral gland

The pharynx in Stilbonematinae consists of an anterior part that is either cylindrical or forms a distinct corpus. When the latter is present, it is followed by a narrow, elongated isthmus. In both cases, the pharynx has a pronounced bulbus at the posterior end (Fig. 1a–j). Its musculature consists of radially arranged myofilaments extending from the ECM surrounding the pharynx to the cuticle lining the central triradiate lumen (Fig. 2a–d). Muscle cells are attached at both ends with hemidesmosomes (Fig. 2a, b, d). As in all nematodes, the pharynx in cross section is divided into one dorsal and two subventral sectors. Each sector is separated from its neighbouring sectors by marginal cells (Fig. 2c). The posterior bulbus harbours a set of gland cells, one in the dorsal and one in each of the subventral sectors, which coincides with the configuration in both Chromadorida and Desmodorida (Chitwood & Chitwood, 1936). Each gland duct leads from the bulbus forward through the pharynx and eventually expands into an ampulla, which opens into the pharynx lumen via the *end apparatus*, which is lined by cuticle (Figs. 1b, 2c, d and 3).

Four sets of muscles are associated with the corpus/anterior pharynx: buccal dilator muscles, anterior and posterior somato-pharyngeal muscles and the spiral muscle. The latter consists of longitudinal muscles which curl around parts of the anterior pharynx and further around the bulbus in various configurations (manuscript in prep.). In the following, the pharynx, its parts and the muscle-configuration of Stilbonematinae species with an enlarged muscular corpus are described, beginning with the corpus, followed by the intermediate isthmus and finally the posterior bulbus. Relative pharynx proportions are summarized in Table 1.

**Table 1:** Comparison of relative pharynx proportion values of investigated species. (avg. isthmus % = average isthmus width in relation to corpus width in %; “ant.”, anterior, “post.”, posterior, the number in parentheses indicates the number of specimens measured)

	*Catanema schiemeri (7)*	*C.sp. 1 (3)*	*Robbea judithae (8)*	*R. lotti (5)*	*R. weberae (6)*	*Cyathorobbea hypermnestra (4)*	*Cy. ruetzleri (5)*	*Cy. agricola (6)*	*Laxus oneistus (6)*
Corpus length %	22–27	26–32	20–27	21–29	20–27	24–33	28–34	24–26	45–52
Corpus length/width	2–3	2–3	2–3	2–3	2–3	1.4–1.7	1.5–2	1.5–2	2–2.7
Isthmus length %	53–59	46–54	59–67	54–61	57–64	35–44	40–58	55–60	18–29
Isthmus length/width	8–13	8–10	11–28	9–24	9–20	3–4	4–8	6–11	2–3
Avg. ant. isthmus %	59	57	39	51	53	66	49	69	54
Avg. post. isthmus %	47	50	35	48	41	63	48	43	58
Avg. bulbus length %	19	21	14	18	16	33	24	18	29

### Corpus

In the genera *Catanema*, *Robbea* and *Cyathorobbea*, the corpus is distinctly set off from the isthmus; a gradual transition is present in *Laxus oneistus*.

#### *Catanema schiemeri* and *C*. sp.

**Fig. 4:**
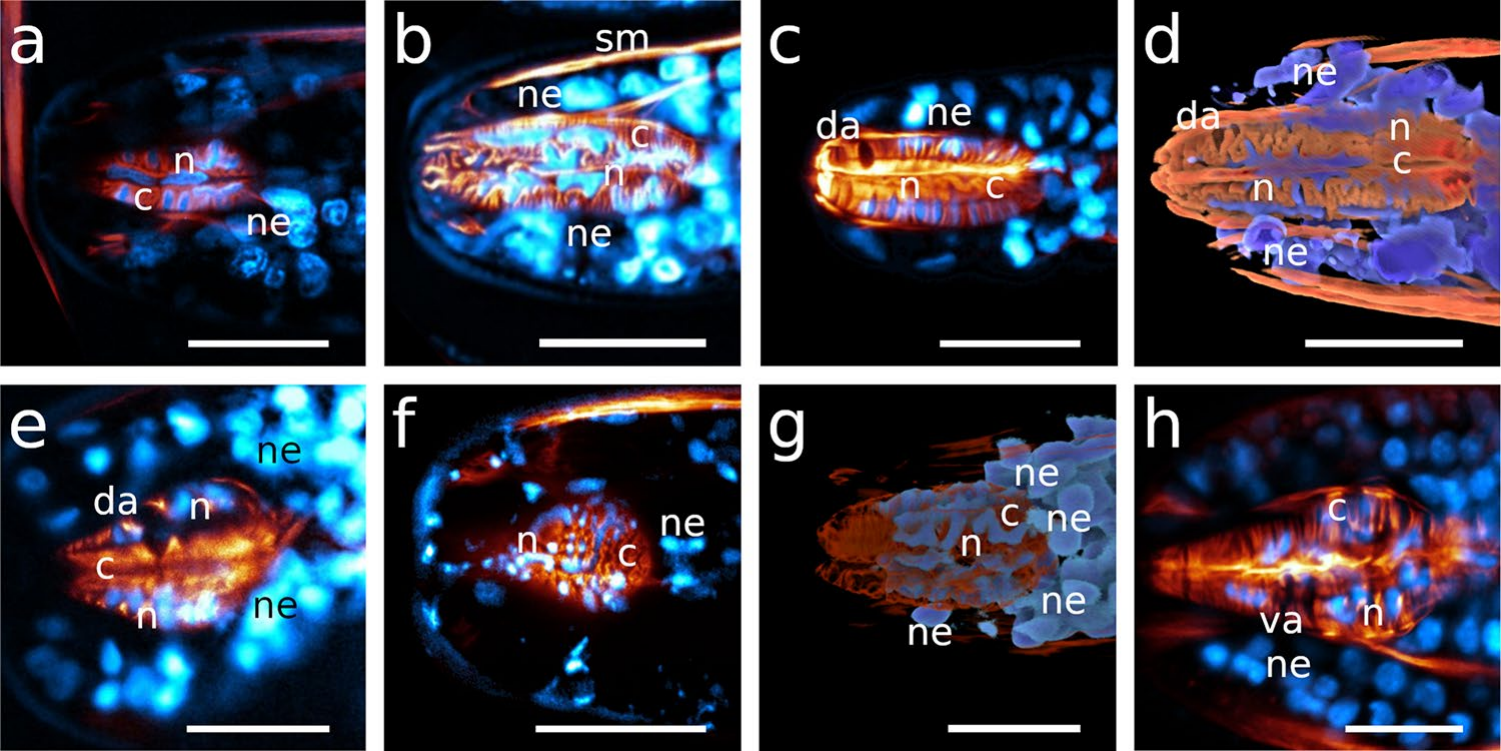
CLSM optical sections showing corpus nuclei. Anterior on the left side. Nuclei inside the orange area are corpus nuclei; nuclei outside this orange background are nuclei of the epidermis **a** *Catanema schiemeri*, **b** *Robbea lotti*, **c** *Robbea judithae*, **d** *Robbea weberae*, volume rendering **e** *Cyathorobbea hypermnestra*, **f** *Cyathorobbea ruetzleri*, **g** *Cyathorobbea agricola*, volume rendering **h** *Laxus oneistus*. Scalebars **a**–**h** 20 μm. c, corpus; da, dorsal ampulla; n, nucleus inside the corpus; ne, epidermal nuclei; sm, somatic muscle; va, subventral ampulla

**Fig. 5:**
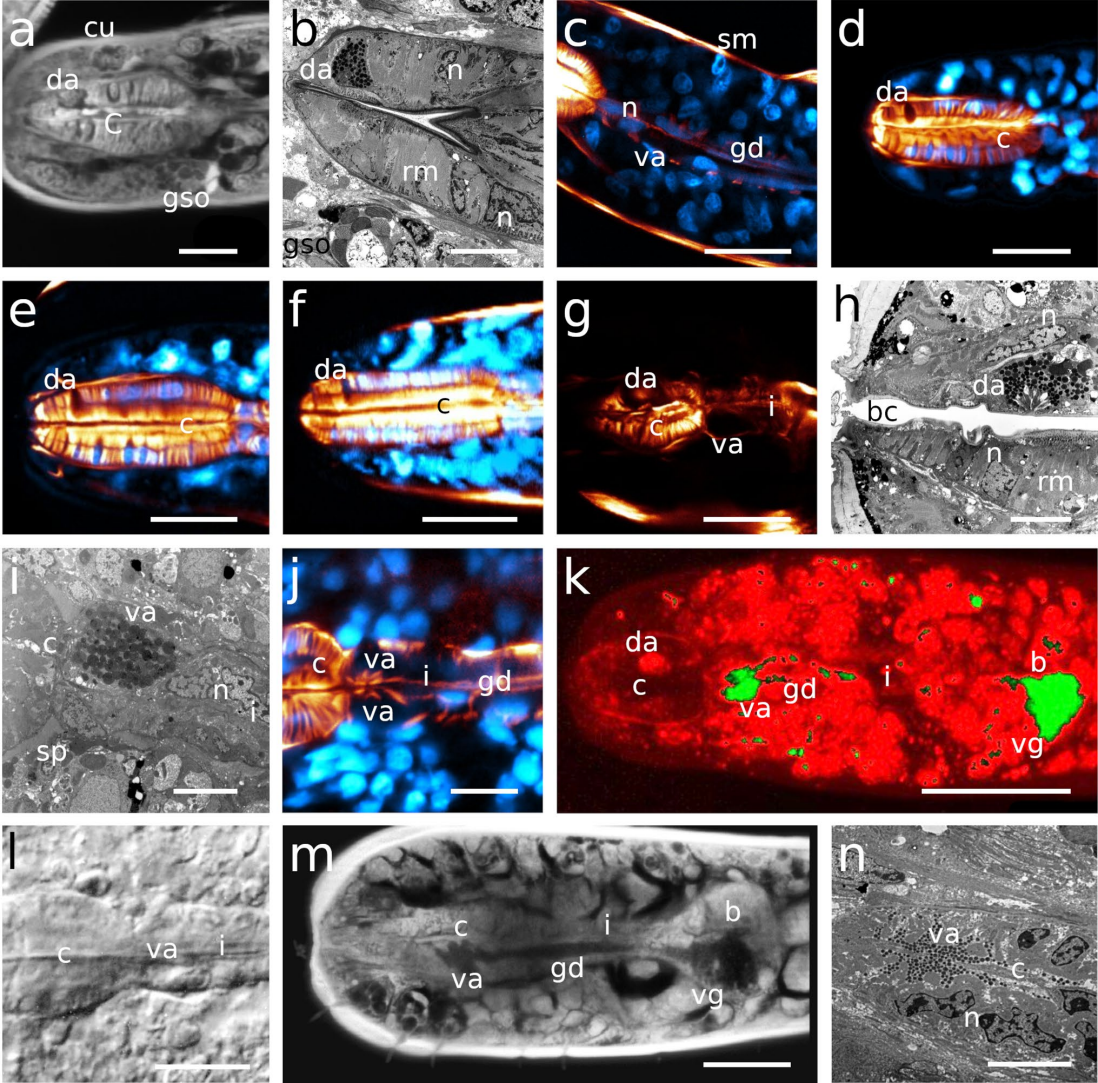
Dorsal and subventral gland ampullae. Anterior on the left side. **a** *Catanema schiemeri*, CLSM optical section, autofluorescence, corpus, **b** *Catanema* *schiemeri*, TEM micrograph, corpus **c** *Catanema* *schiemeri*, CLSM micrograph, isthmus, musculature in orange, nuclei in blue **d** *Robbea lotti*, CLSM optical section, musculature in orange, nuclei in blue, corpus **e** *Robbea weberae*, CLSM optical section, musculature in orange, nuclei in blue, corpus **f** *Robbea judithae*, CLSM optical section, musculature in orange, nuclei in blue, corpus **g** *Cyathorobbea hypermnestra*, CLSM optical section, musculature in orange, corpus **h** *Cyathorobbea hypermnestra*, TEM micrograph, corpus **i** *Cyathorobbea hypermnestra* TEM micrograph, anterior isthmus **j** *Cyathorobbea ruetzleri*, CLSM optical section, musculature in orange, nuclei in blue, anterior isthmus **k** *Cyathorobbea agricola*, CLSM optical section, autofluorescence, autofluorescence (UV laser) of gland secretion in green, pharynx region **l** *Cyathorobbea agricola*, optical section, corpus-isthmus transition, subventral ampulla **m** *Laxus oneistus*, CLSM optical section, autofluorescence, pharynx region **n** *Laxus oneistus*, TEM micrograph, subventral ampulla, corpus. Scalebars **a** 10 μm, **b** 5 μm,**c** 20 μm, **d** 15 μm, **e** 15 μm, **f** 15 μm, **g** 20 μm, **h** 5 μm, **i** 5 μm, **j** 10 μm, **k** 20 μm, **l** 10 μm, **m** 20 μm, **n** 5 μm. bc, buccal cavity; c, corpus; cu, cuticle; da, dorsal ampulla; gd, gland duct; gso, glandular sensory organ; i, isthmus; n, nucleus; rm, radial musculature; sm, somatic muscle; sp, spiral muscle; va, subventral ampulla; vg, subventral gland

**Fig. 6:**
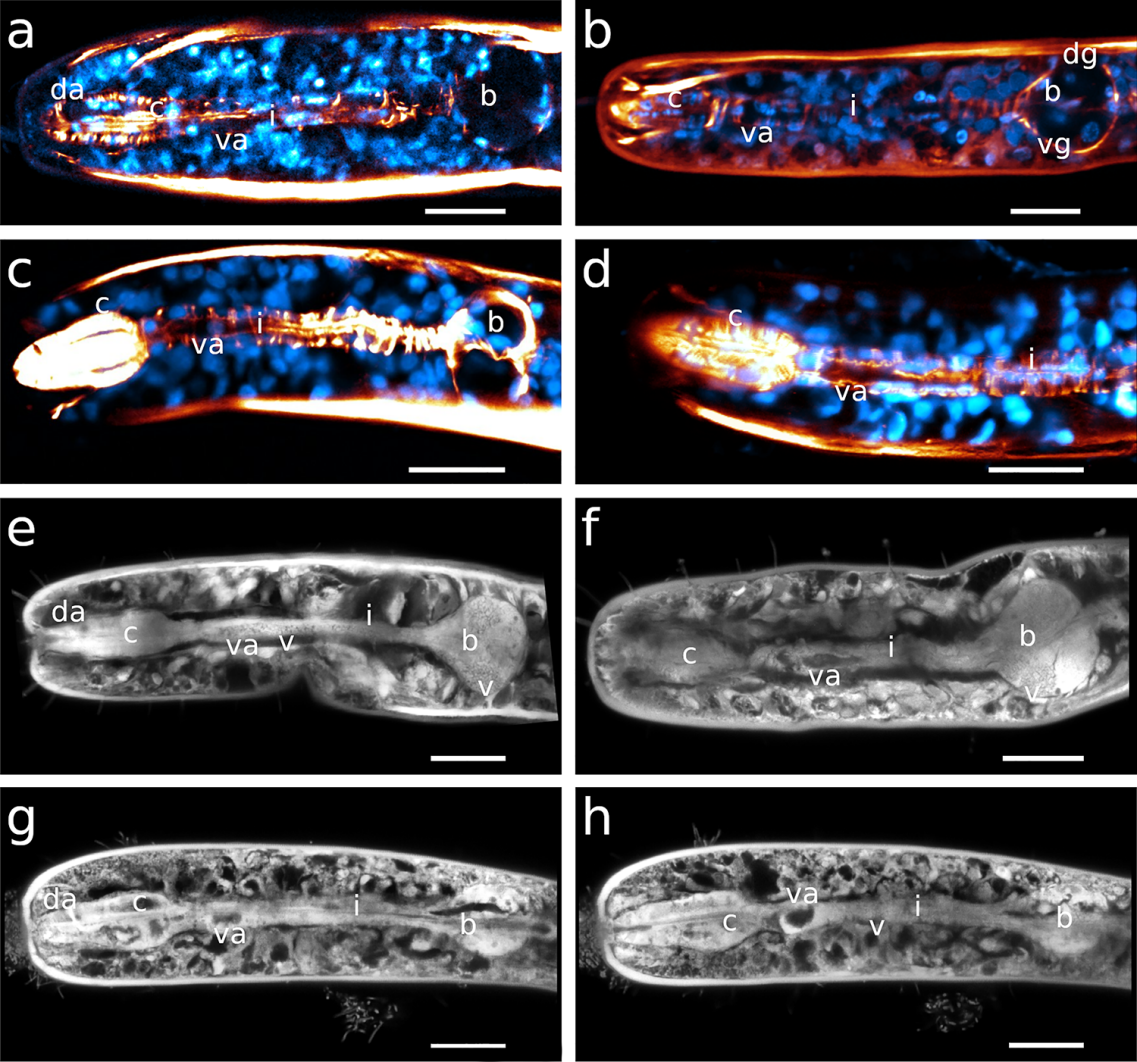
Pharyngeal region, CLSM optical sections, *Catanema schiemeri*, dorsal and subventral ampullae, musculature in orange, nuclei in blue **a** lateral view, **b**–**d** subventral view. **e**–**h** *Catanema* sp., autofluorescence optical sections, **e** specimen 1, **f** specimen 2, **g**–**h** specimen 3. Scalebars 20 μm. b, bulbus; c, corpus; da, dorsal ampulla; dg, dorsal gland; i, v, gland vesicles; va, subventral ampulla; vg, subventral gland

The egg-shaped corpus tapers distinctly towards the anterior end in both species. Myofilaments are very densely arranged, with their angles changing gradually throughout the corpus, pointing in a forward direction anterior-most, approximately perpendicular in the centre of the corpus and gradually assuming a posterior direction. The myoepithelium of the corpus bears basally located nuclei that distort the course of the myofilaments (Table 1; Figs. 4a, 5b, and 6a, b).

#### *Robbeajudithae*, *R. lotti* and *R. weberae*

**Fig. 7:**
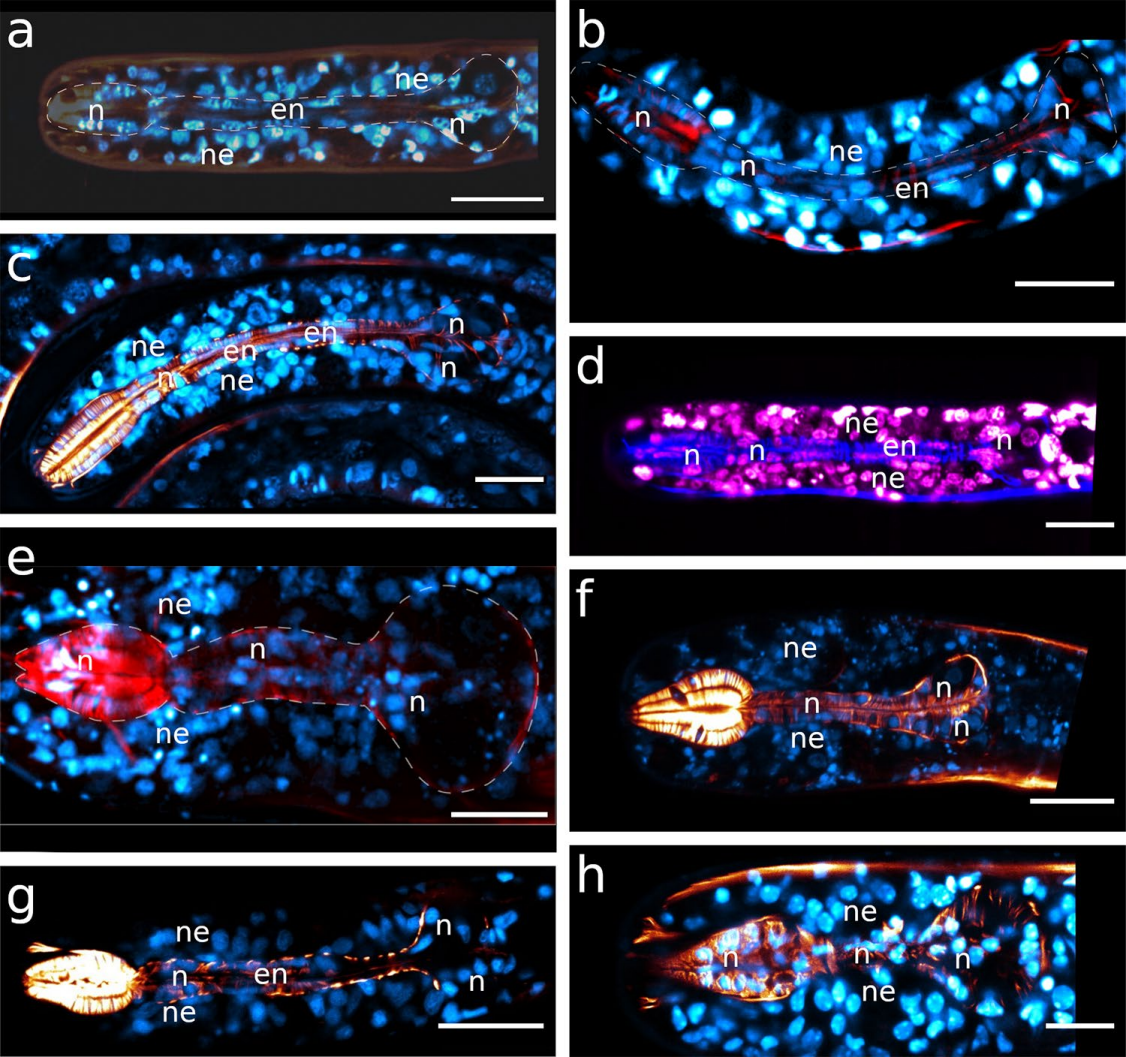
Pharyngeal region, pharynx nuclei, CLSM optical sections. Colors: a–c, e, f: musculature in red/orange, nuclei in blue; d: musculature in blue, nuclei in magenta; nuclei within the orange labelled area/outline are pharynx nuclei. For some species the outline of the pharynx is further indicated with white dashed lines. **a** *Catanema schiemeri*, **b** *Robbea judithae*, **c** *Robbea lotti*, **d** *Robbea weberae*, **e** *Cyathorobbea hypermnestra*, **f** *Cyathorobbea ruetzleri*, **g** *Cyathorobbea agricola*, **h** *Laxus oneistus*. Dashed lines indicate the outline of the pharynx. Scalebars 20 μm. en, elongated nucleus inside the pharynx; n, nucleus inside the pharynx; ne, epidermal nuclei

**Fig. 8:**
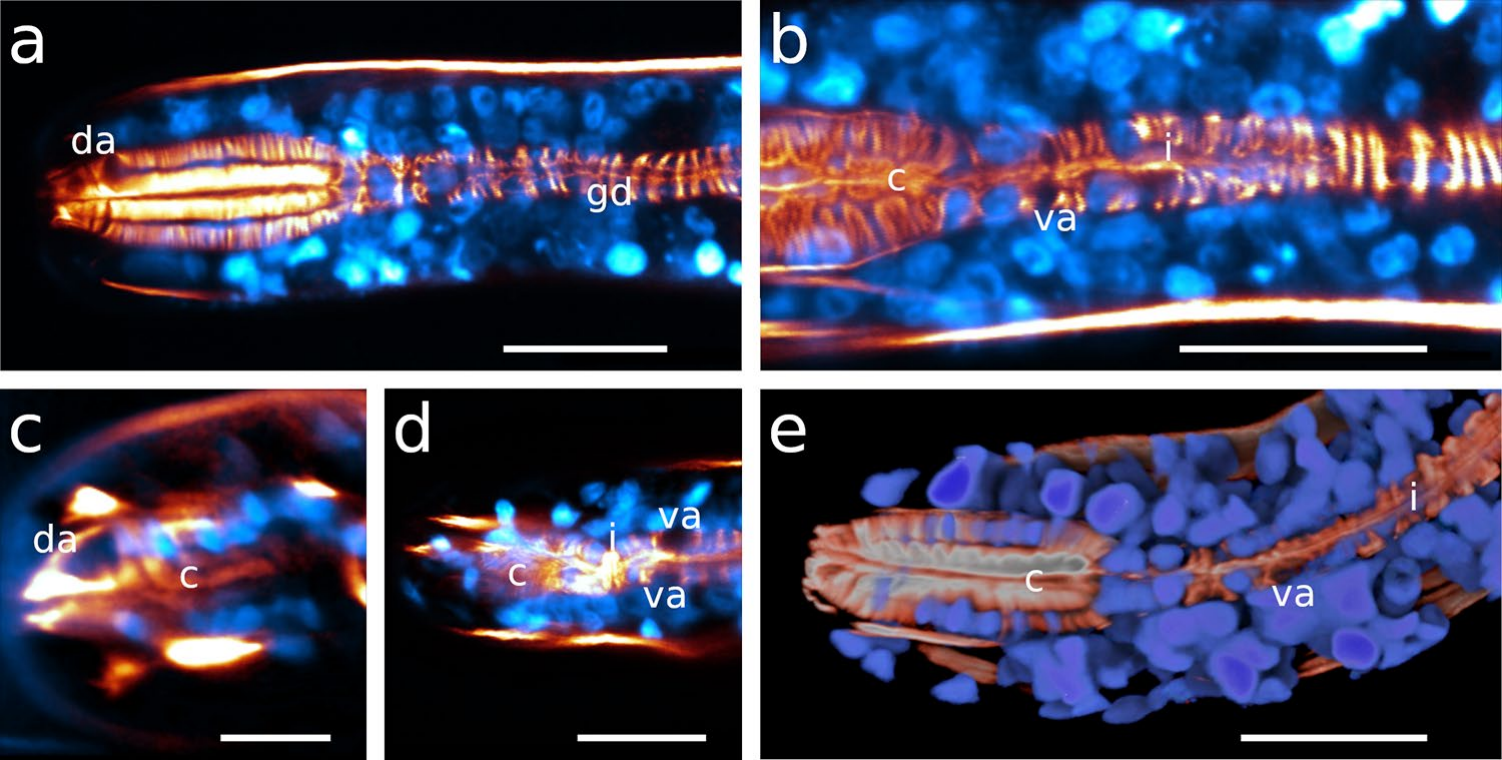
Pharyngeal region, CLSM optical sections, dorsal and subventral ampullae, musculature in orange, nuclei in blue **a**, **b** *Robbea weberae*, **c**, **d** *Robbea lotti*, **e** *Robbea judithae*. Scalebars **a**, **b**, **d**, **e** 20 μm; **c** 10 μm. c, corpus; da, dorsal ampulla; gd, gland duct; i, isthmus; va, subventral ampulla

**Fig. 9:**
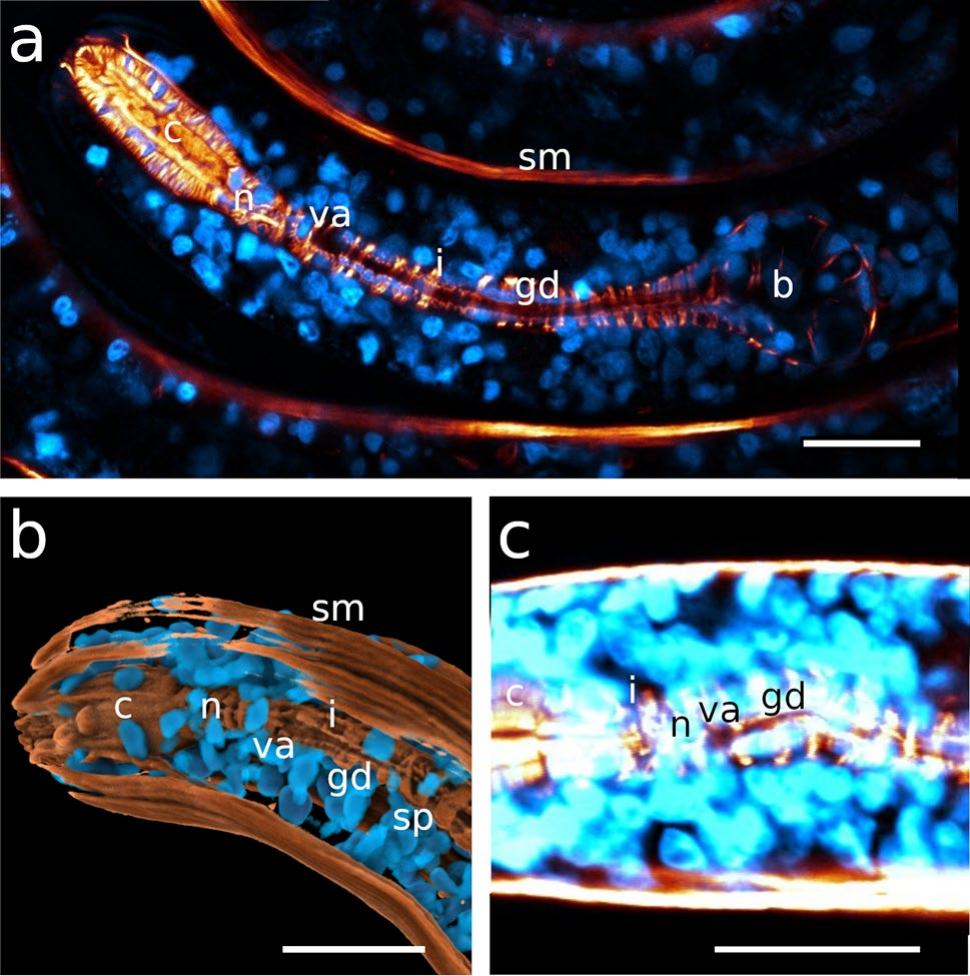
Pharyngeal region, subventral ampullae. Musculature in orange, nuclei in blue **a** *Robbea lotti*, CLSM optical section **b** *Robbea judithae*, volume rendering **c** *Robbea weberae*, CLSM optical section. Scalebars 20 μm. b, bulbus; c, corpus; gd, gland duct; i, isthmus; n, nucleus; sm, somatic muscle; sp, spiral muscle; va, subventral ampulla

**Fig. 10:**
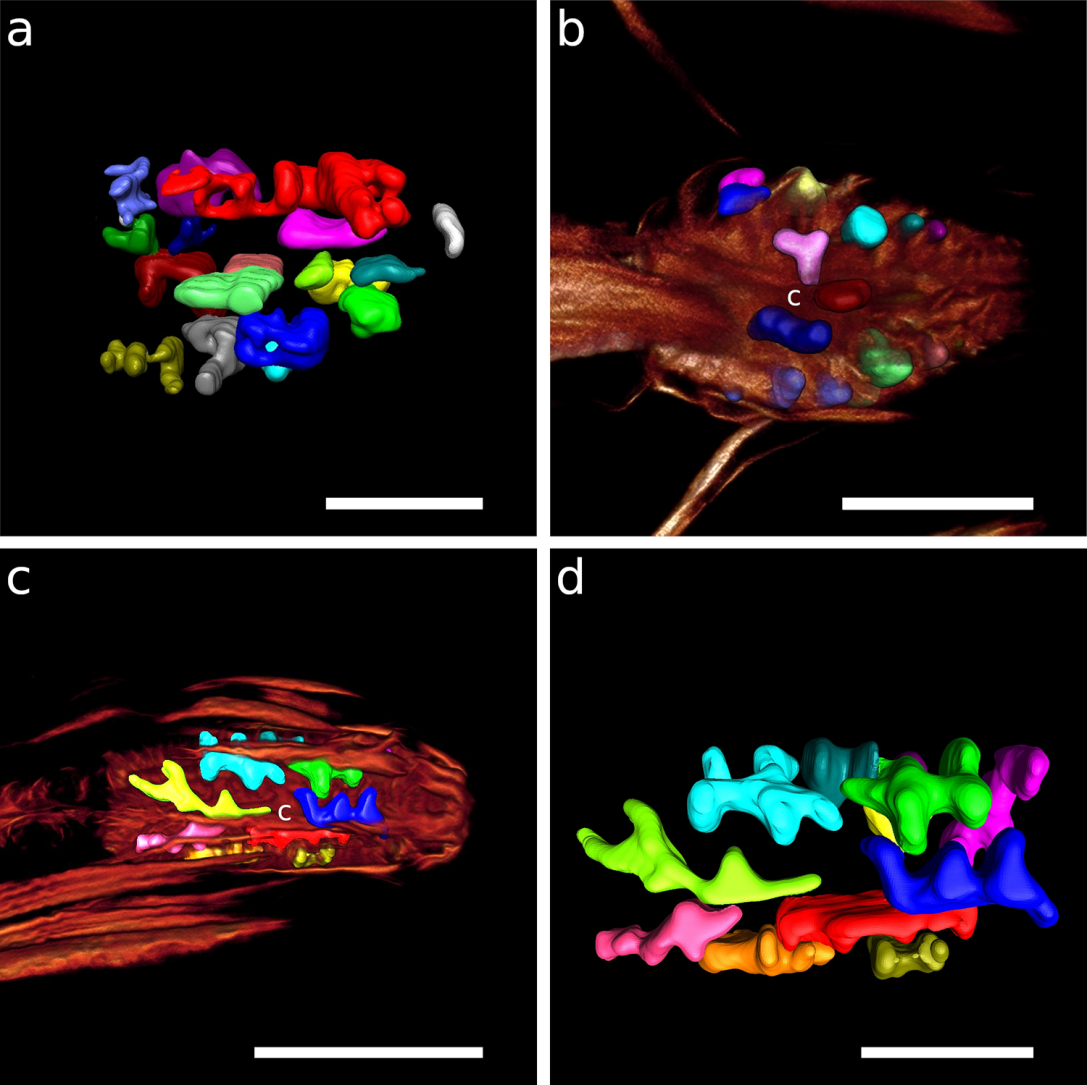
3D Reconstructions of corpus nuclei, anterior on the right **a** *Catanema schiemeri*, **b** *Cyathorobbea hypermnestra*, **c**, **d** *Robbea judithae*. Scalebars **a** 10 μm, **b** 15 μm, **c** 20 μm, **d** 10 μm. c, corpus

The corpus is an ellipsoid, only very slightly tapering towards the anterior end. The orientation of the myofilaments is similar in all three species, being directed forward at the anterior end, changing gradually to perpendicular at mid-length and backward directed at the posterior end (Fig. 1d, e; Fig. 4d; Fig. 5f; Fig. 7h, Fig. 8a). Myofilaments are displaced at several positions providing space for nuclei in all three species (Figs. 4b, d, 5d–f, 7c, d, 8c, e, 9a, 10c).

#### *Cyathorobbea hypermnestra*, *Cy. ruetzleri* and *Cy. agricola*

**Fig. 11:**
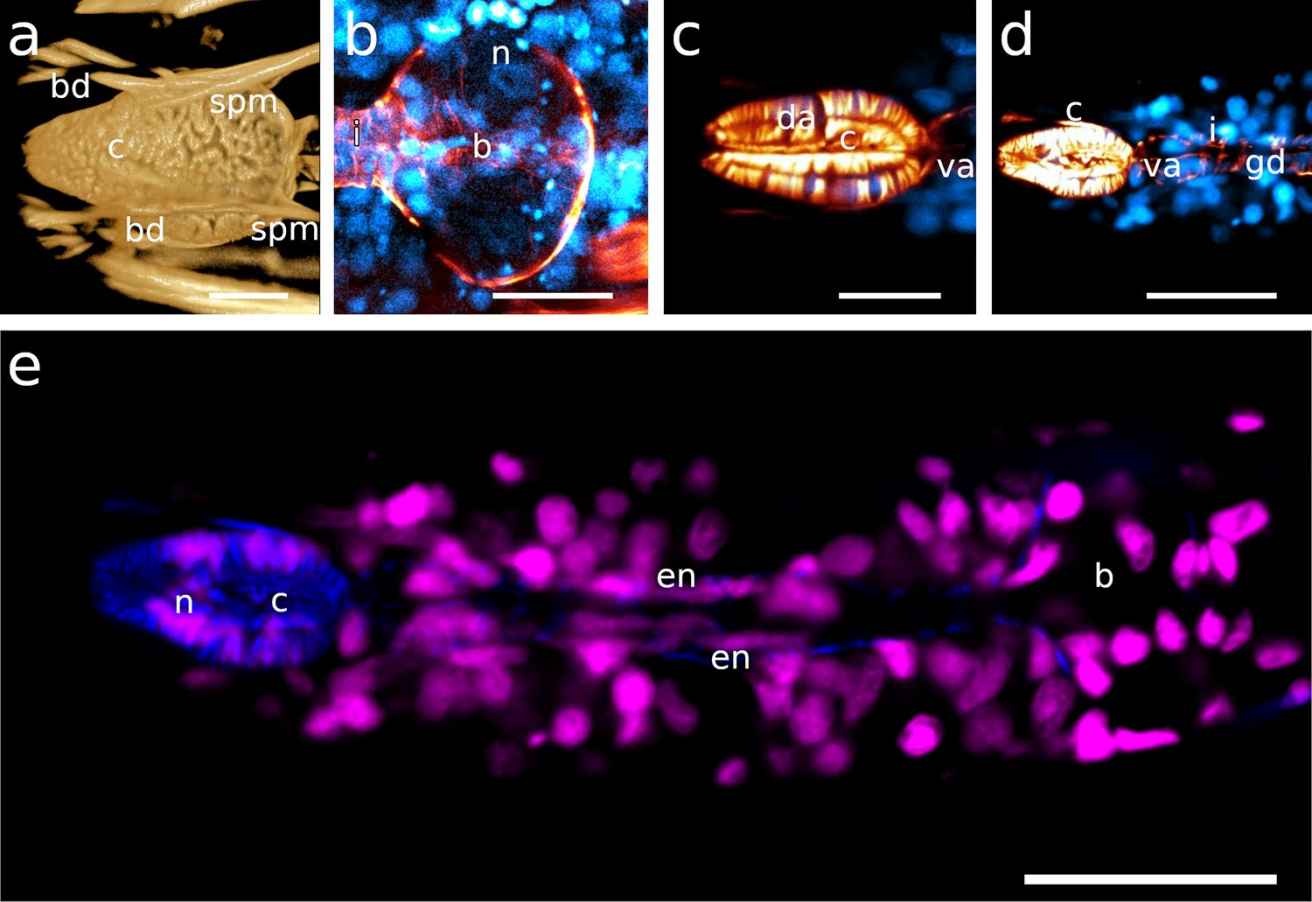
Pharyngeal region **a** *Cyathorobbea ruetzleri*, volume rendering, corpus musculature **b** *Cyathorobbea hypermnestra*, CLSM maximum intensity projection, bulbus, musculature in orange, nuclei in blue **c**, **d** *Cyathorobbea agricola*, CLSM optical sections, corpus region, musculature in orange, nuclei in blue **e** *Cyathorobbea agricola*, CLSM optical section, pharynx region, musculature in blue, nuclei in magenta. Scalebars **a** 10 μm, **b** 20 μm, **c** 10 μm, **d** 20 μm, **e** 20 μm. b, bulbus; bd, buccal dilator; c, corpus; da, dorsal ampulla; en, elongated nucleus; gd, gland duct; i, isthmus; n, nucleus; spm, somato-pharyngeal muscle; va, subventral ampulla

The corpus is pyriform and tapers towards the anterior end. It showed the strongest F-actin signal of the whole pharynx. The myofilament orientation is similar in all three species: directed forward at the anterior-most end, becoming gradually perpendicular towards the second third of the corpus and then directed noticeably backward at the posterior end of the corpus (Figs. 1d, g–i, 4e, 5d, 7e, 11c, d). Basal nuclei are placed in between the very densely arranged myofilaments on the corpus of all three species (Figs. 4e–g, 10b and 11c).

#### *Laxusoneistus*

The corpus has a vase-like shape and is about two to three times as long as wide, tapering gradually over a longer distance towards the anterior end. It is conspicuously large compared to the corpus of species in other genera and constitutes around 45–52% of the pharynx length (Table 1). The myofilaments are only slightly directed forward and the anterior-most end and nearly perfectly perpendicular until 2/3 of the corpus length. From this point on, they are oriented slightly backwards. The density of myofilaments and intensity of the F-actin signal were equal in both corpus and the succeeding isthmus. Basally situated nuclei are positioned in between myofilaments in the corpus (Figs. 1j, 4h, 5n and 7h).

### Isthmus

The isthmus is surrounded by the circumpharyngeal nerve ring, the central nervous system. In all species, the myofilaments are perpendicular to the longitudinal axis of the isthmus throughout its entire length. The latter decreases in width from anterior to posterior in all investigated species except for *Cyathorobbea ruetzleri*, where the decrease is only marginal, and *Laxus oneistus*, where the width increases slightly (Table 1).

#### *Catanemaschiemeri* and *C*. sp.

The isthmus is slender in both species and tapers the most in *C. schiemeri* out of all investigated species (Table 1). The labelled myofilaments of *C*. *schiemeri* are regularly arranged and displaced distinctly only at positions of the gland ampullae and gland ducts (Figs. 1c, 4a, c and 6c, d).

#### *Robbeajudithae*, *R. lotti* and *R. weberae*

The isthmus of this genus is more slender than in any other investigated species and constitutes the largest amount of the pharynx length (Fig. 1e, f; Table 1; Figs. 7c, d and 9a).

#### *Cyathorobbea hypermnestra*, *Cy. ruetzleri* and *Cy. agricola*

The isthmus is short and stout in *Cy*. *hypermnestra* and *Cy*. *ruetzleri*, but elongated and narrow in *Cy*. *agricola*. In the latter, it also tapers the strongest from anterior to posterior compared to all other investigated species (Fig. 1g–i; Table 1; Fig. 7f–h).

#### *Laxusoneistus*

**Fig. 12:**
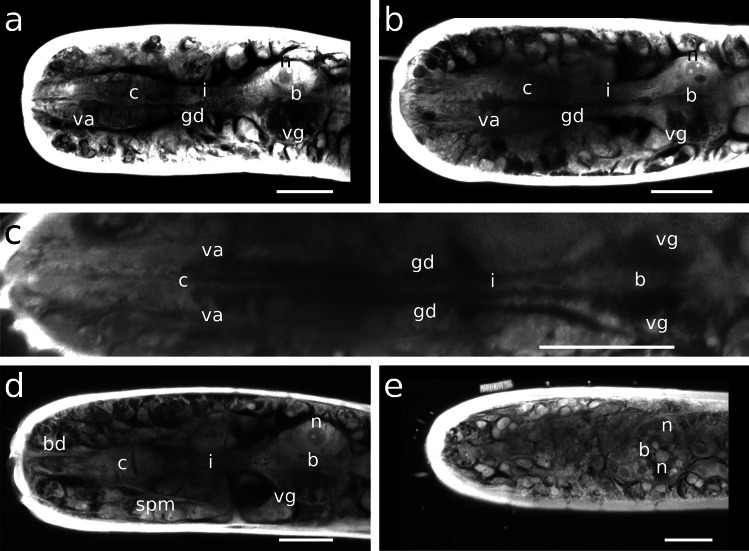
Pharynx region of *Laxus oneistus*, CLSM optical sections, autofluorescence **a**, **b** subventral view, **c** ventral view, **d** dorso-lateral view, **e** ventral view. Scalebars 20 μm. b, bulbus; bd, buccal dilator; c, corpus; gd, gland duct; i, isthmus; n, nucleus; spm, somato-pharyngeal muscle; va, subventral ampulla; vg, subventral gland

Out of all other investigated species, the isthmus of *Laxus oneistus* is the shortest and stoutest, accounting only for less than 30% of the total pharynx length; it slightly increases in width from anterior to posterior (Fig. 1j; Table 1; Fig. 12a–c).

### Bulbus

The isthmus-bulbus transition is gradual in all investigated species.

#### *Catanemaschiemeri* and *C*. sp.

**Fig. 13:**
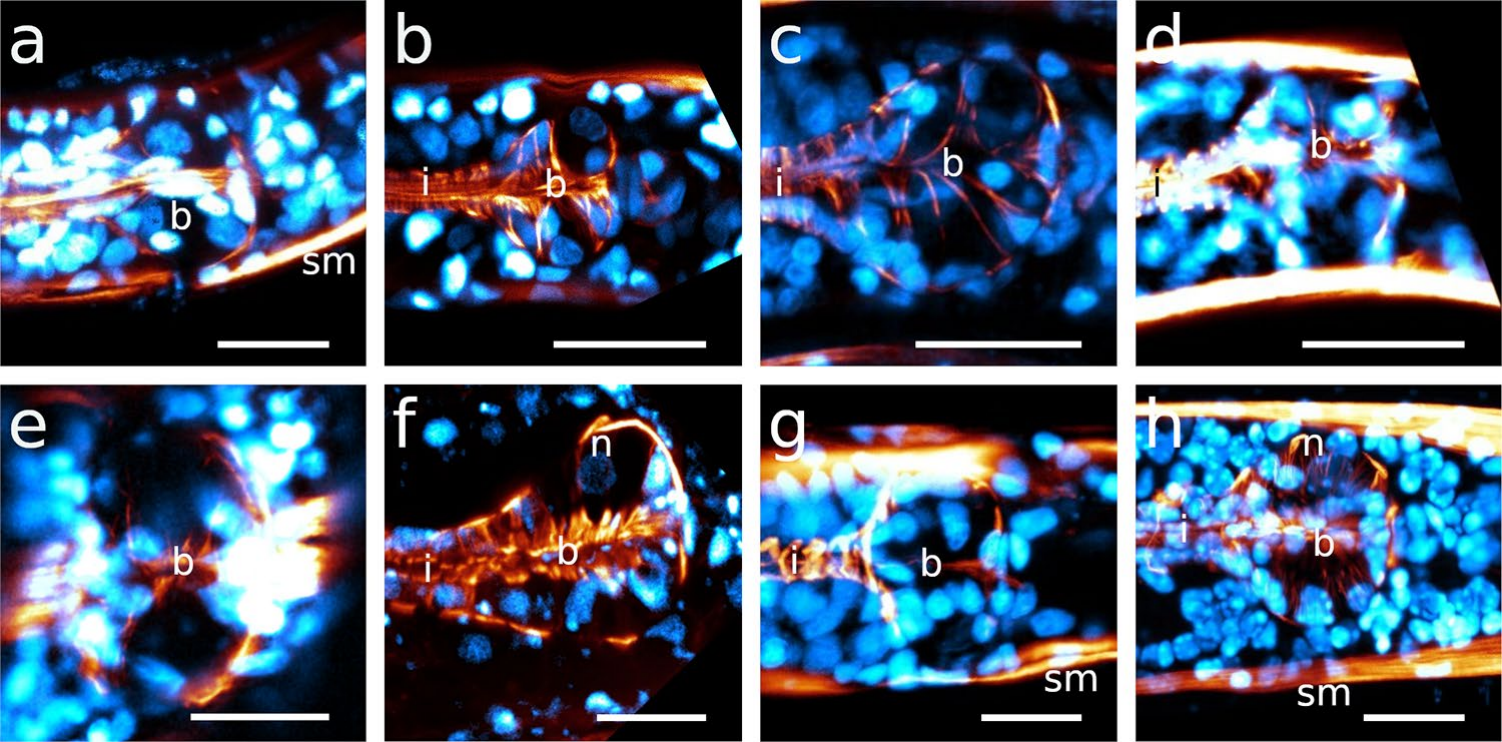
Posterior pharynx region. CLSM maximum intensity projections, musculature in orange, nuclei in blue. **a** *Catanema schiemeri*, **b** *Robbea judithae*, **c** *Robbea lotti*, **d** *Robbea weberae*, **e** *Cyathorobbea hypermnestra*, **f** *Cyathorobbea ruetzleri*, **g** *Cyathorobbea agricola*, **h** *Laxus oneistus*. Scalebars **a**–**e**, **h** 20 μm; **f**, **g** 10 μm. b, bulbus; i, isthmus; n, nucleus; sm, somatic muscle

The spherical to slightly heart-shaped posterior bulbus accounts for roughly the same amount of the pharynx total length and is mainly devoid of musculature in both species (Fig. 1c; Table 1; Figs. 6a–c, e–h and 13a).

#### *Robbeajudithae*, *R. lotti* and *R. weberae*

The posterior bulbus is the smallest in *R*. *judithae*, slightly larger in *R*. *weberae* and largest in *R*. *lotti*. It is wider than long in both *R*. *judithae* and *R*. *weberae* and nearly spherical in *R*. *lotti* (Table 1). The subventral portion is slightly more prominently developed in the latter species (Figs. 1d, 9a and 13c). Myofilaments are limited to the anterior and posterior part in *R*. *judithae* and *R*. *weberae* (Figs. 1e, f and 13b, d) and evenly distributed in *R*. *lotti* (Figs. 1d and 13c).

#### *Cyathorobbea hypermnestra*, *Cy. ruetzleri* and *Cy. agricola*

The bulbus is wider than long in both *Cy*. *hypermnestra* and *Cy*. *agricola* and is distinctly asymmetrical in *Cy*. *ruetzleri*, i.e. with a more prominent dorsal part featuring a triangular shape in the sagittal section. The bulbus of *Cy*. *hypermnestra* is the largest compared to all other investigated species (Fig. 1g–i; Table 1). Within the genus, *Cy*. *agricola has* the smallest bulbus (Fig. 1g–i; Table 1). In *Cy*. *hypermnestra* the radial myofilaments are delicate and restricted to the anterior and posterior third of the bulbus (Figs. 1g and 13b). In *Cy*. *ruetzleri*, delicate myofilaments are slightly more present in the subventral sectors whereas in the dorsal sector, they are restricted to the anterior and posterior-most part (Figs. 1h, 7f and 13f). In contrast, myofilaments in *Cy*. *agricola* are absent in the anterior part of the bulbus and are restricted to the posterior-most end (Figs. 1i, 7d and 13g).

#### *Laxusoneistus*

The bulbus is nearly spherical and the second largest in relation to the pharynx length compared to all other investigated species (Table 1). In the dorsal sector, the radial myofilaments are evenly arranged and only noticeably displaced by one conspicuously large nucleus (Figs. 1j, 12a, b, d and 13h). Each subventral sector has a distinct zone devoid of myofilaments (Figs. 1j and 12a-c, e). Nuclei are surrounded by evenly arranged myofilaments (Figs. 7h and 13h).

### Gland ampullae

#### *Catanema schiemeri* and *C*. sp.

In both *C. schiemeri* and *C.* sp., the dorsal gland ampulla is positioned at the anterior end of the corpus (Figs. 5a, b and 6a, e, g). Both subventral gland ampullae are situated near the anterior end of the isthmus, slightly posterior to the anterior-most nuclei of the isthmus in the respective subventral sectors (Figs. 5c, 6a–h and 7a).

#### *Robbeajudithae*, *R. lotti* and *R. weberae*

The ampulla in the dorsal sector is positioned at the anterior end of the corpus in all three species (Figs. 4c, d, 5d–f and 8a, c). The ampullae at the end of the subventral gland ducts open into the isthmus at a short distance from the posterior end of the corpus, *slightly posterior to the anterior-most nuclei* of the isthmus (Figs. 8b, d, e and 9a–c).

#### *Cyathorobbea hypermnestra*, *Cy. ruetzleri* and *Cy. agricola*

The dorsal gland opening is positioned roughly at the centre of the corpus in all three species, at around 40–50% of the corpus length (Figs. 1d, 2c, d, 4e, 5g, h, k and 11c). The subventral gland ampullae also have the same position in all three species: at the anterior-most end of the isthmus, *anterior to the anterior-most nuclei* of the isthmus (Figs. 5i–l and 11c, d).

#### *Laxusoneistus*

Neither a dorsal gland nor a dorsal gland ampulla was observed in this species. The subventral gland ampullae are positioned *in the corpus* at roughly 50% of its length, anterior to the position of maximum corpus width (Figs. 5m, n and 12a–c).

### Cell nuclei

#### *Catanemaschiemeri*

The nuclei in the corpus are strongly lobulated (Fig. 10a) and fit in-between muscle fibres (Fig. 4a. The narrow isthmus contains both ovoid and strongly elongated nuclei; the former is located in the anterior and posterior part of the isthmus (in some individuals the posterior-most isthmus is exceptionally slender and lacks nuclei); the elongated nuclei extend along the mid part of the isthmus (Figs. 6a–d and 7a). Nuclei in the posterior bulbus are ovoid to spherical and are located both close to the lumen and the distal margin of the bulbus (Figs. 6a–c, 7a and 13a).

#### *Robbeajudithae*, *R. lotti* and *R. weberae*

The corpus of all three species exhibits strongly lobulated nuclei fitting in between myofilaments (Figs. 5b–d and  10c, d). In the isthmus, their shapes vary from ovoid to spherical in the anterior part, distinctly elongated in the centre and ranging from ovoid to slightly elongated in the posterior part (Figs. 7b–d, 8a, b, e and 9a–c). The nuclei in the bulbus are spherical to ovoid and are located at the distal margin in *R*. *judithae* (Figs. 7b and 13b), at the distal margin and close to the bulbus lumen in *R*. *lotti* (Figs. 7c, 9a and 13c) and are regularly distributed in *R. weberae* (Figs. 7d and 13d).

#### *Cyathorobbea hypermnestra*, *Cy. ruetzleri* and *Cy. agricola*

Nuclei of the corpus are ovoid to slightly elongated and lobulated in both *Cy. hypermnestra* and *Cy. ruetzleri* to fit in between myofilaments and the dorsal ampulla (Figs. 4e, f, 5h and 10b). *Cy. agricola* has elongated nuclei in the corpus that show a peculiar zigzag pattern (Fig. 4g). In the isthmus and posterior bulbus, nuclei are ovoid to spherical in both *Cy*. *hypermnestra* and *Cy*. *ruetzleri* (Figs. 5i, 7e, f and 13e, f). In the isthmus of *Cy*. *agricola*, their shape ranges from ovoid to elongated. The latter nuclei are roughly four times longer than wide (Figs. 7g and 11e). Some individuals lack nuclei in the posterior-most isthmus due to its slenderness. In both *Cy. hypermnestra* and *Cy*. *ruetzleri*, one spherically shaped nucleus in the dorsal sector of the bulbus is slightly larger than the others (Figs. 11b and 13f), whereas the ovoid nuclei in the bulbus of *Cy*. *agricola* are roughly of equal size (Figs. 7g, 11e and 13g).

#### *Laxus oneistus*

The corpus nuclei are either elongated and bent or lobulated (Figs. 4h, 5n and 7h). Throughout the isthmus, nuclei are ovoid and regularly distributed (Fig. 7h). In addition to three especially prominent nuclei in the dorsal and subventral sectors of the bulbus (Figs. 12a, b, d, e and 13h), other smaller nuclei are positioned at the distal margin and close to the bulbus lumen (Figs. 7h and 13h).

## Discussion

With the emergence of molecular phylogenies, the classification system of Nematoda has been recently updated using molecular data, and morphological phylogenies were used for those taxa for which molecular data are lacking (De Ley & Blaxter, 2002); Schmidt-Rhaesa, 2014). For the discussion, we use the reference database “Nemys – The World Database of Nematodes.” Since a taxonomic database is always a work in progress, it was archived on 28.02.2023, and the archived version can be opened via the following link: https://web.archive.org/web/20230228125625/https://nemys.ugent.be/.

### Pharynx types in nematodes

The pharynges of nematodes are highly diverse in structure and function. An early attempt to group them into several pharynx types was based on their general appearance, inner morphology and on the taxa for which the specific type was characteristic (Filipjév & Stekhoven, 1941). Later, nematode pharynges were classified into three basic types: one-part, two-part and three-part (Allen, 1960). Recent works combine both approaches (Bird & Bird, 1991; Maggenti, 1981).

#### One-part pharynx

A simple one-part pharynx type is present in Enoplida Filipjév, 1929 (Nemys eds., 2024) (e.g. Enoplina, Ironina, Tripyloidina, Trefusiina, Oncholaimina), Triplonchida Cobb, 1919 (e.g. Tripylina, Tobrilina), Mononchida Jairajpuri, 1969 and in other Enoplea that possess a completely cylindrical pharynx without pronounced subregions.

#### Two-part pharynx

A two-part pharynx has a cylindrical, narrow anterior part and the posterior part is either a spherical bulb or an elongated cylinder. The bulbous type is characteristic of Chromadorida and Desmodorida (Tchesunov, 2014a, b). A two-part pharynx with cylindrical posterior part is present in several plant parasitic genera such as *Xiphinema*, *Longidorus* (both Dorylaimia), *Trichodorus* (Enoplia), *Spirura* (Rhabditida, Spiruromorpha, Spiruridae), *Heliconema* and *Physaloptera* (both Spiruromorpha, Physalopteridae).

#### Three-part pharynx

**Fig. 14:**
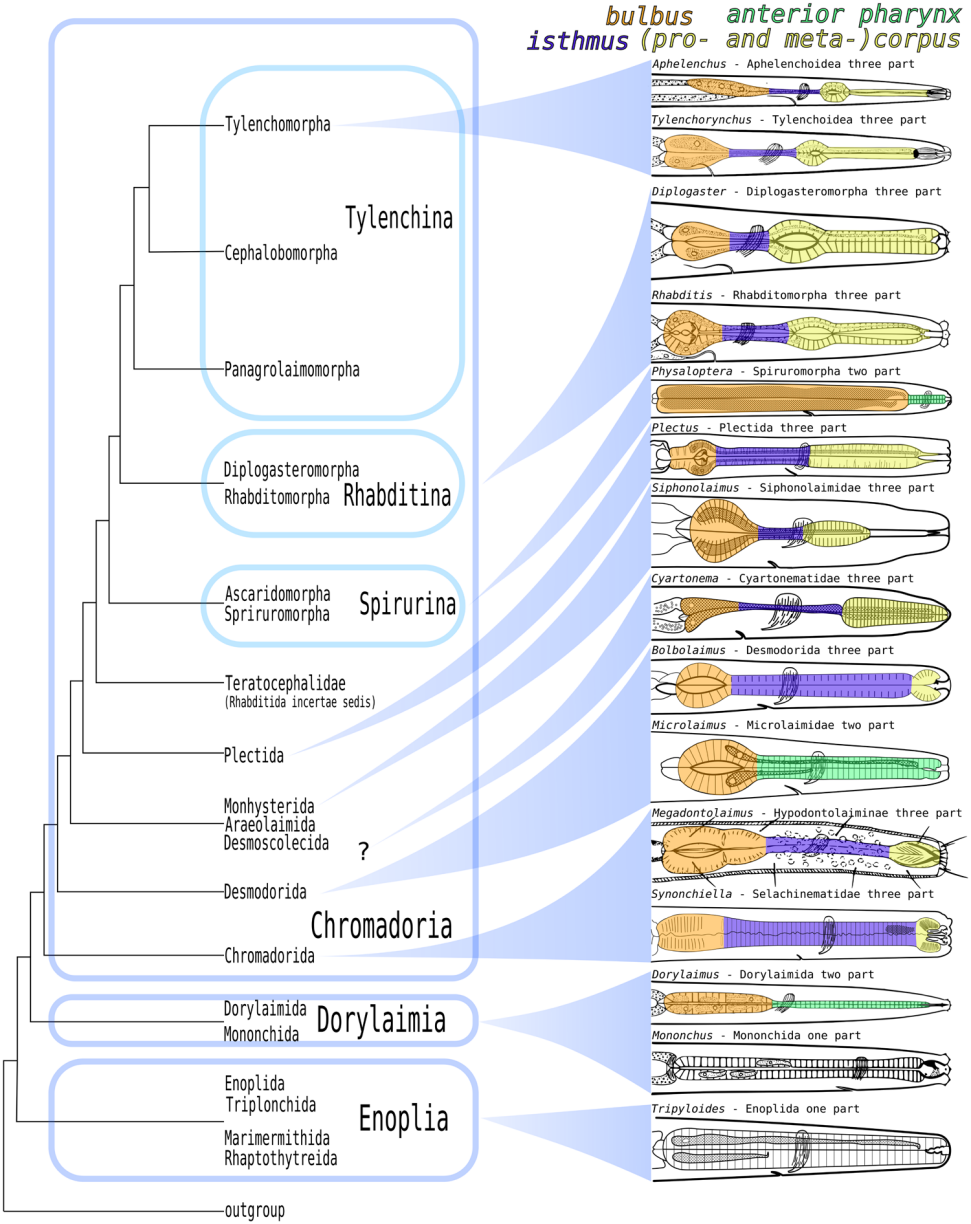
Pharynx types across Nematoda. Systematic modified from  Smol et al. (2020). Pharynx drawings of Aphelenchoidea, Tylenchoidea, Diplogasteromorpha, Rhaditomorpha, Dorylaimida and Mononchida modified from Hirschmann (1960), of Spiruromorpha modified from both Chitwood and Chitwood (1977) and Fu et al. (2018), of Plectida modified from Holovachov (2004), of *Siphonolaimus* modified from Ott (1972), of *Cyartonema* modified from Tchesunov (1989), of *Bolbolaimus* modified from Cobb (1920) and Riemann (1966), of *Microlaimus* modified from Chitwood and Chitwood (1977), of *Megadontolaimus* modified from Timm (1969), of *Synonchiella* modified from Ott (1972), of *Tripyloides* modified from Chitwood and Chitwood (1977)

A three-part pharynx is found within Chromadoria either characteristic of a whole higher taxon or in conjunction with special structures (teeth, jaws, spears) on the family or even genus level. It is characterized by three regions: the anterior corpus, the intermediate isthmus and the posterior bulbus (Fig. 14).

The only three-part pharynx outside Chromadoria has been reported in Aulolaimoididae (Dorylaimina, Tylencholaimoidea). It has a cylindrical anterior part, a metacorpus-like pyriform swelling and a short, distinctly set-off isthmus surrounded by gland tissue and a valved, pyriform bulbus (Andrassy, 2009).

Several three-part pharynx types inside Chromadoria were distinguished by Maggenti (1981). The “diplogasteroid” and “tylenchoid” three-part pharynx types are very similar. In both, the corpus is subdivided into a pro- and metacorpus. The metacorpus has a very prominent musculature and is equipped with well-sclerotized luminal walls, the “crescentic valve” (Maggenti, 1981; Subbotin, 2014) and is the main pumping structure. Both in Diplogasteromorpha (Rhabditida, Rhabtidina) and Tylenchomorpha (Rhabditida, Tylenchina), the musculature in the posterior bulb is reduced while at the same time, the gland cells are conspicuous and in Aphelenchoidea and Tylenchoidea (both Tylenchomorpha) may even overlap with the intestine (Decraemer et al., 2014; Hirschmann et al., 1960; Maggenti, 1981). The pharynges of these two groups differ in the position of the dorsal gland orifice: at the anterior end of either the metacorpus (Aphelenchoidea) or the procorpus (Tylenchoidea)(Hirschmann et al., 1960).

In the “rhabditoid” pharynx, which is present within Rhabditomorpha (Rhabditida, Rhabditina), the corpus is subdivided into a pro- and metacorpus (the latter being markedly swollen). The pharyngeal tubes (sensu Holovachov, 2004) are well developed in the procorpus (Albertson & Thompson, 1976; Maggenti, 1981), absent in the anterior metacorpus and present again in the posterior metacorpus and in the anterior end of the isthmus (Albertson & Thompson, 1976). The isthmus is characterized by its lack of nuclei (Albertson & Thompson, 1976; Chitwood & Chitwood, 1977; Maggenti, 1981), and the posterior bulb houses a prominent grinding structure, the “butterfly valves” (Fürst von Lieven, 2003; Maggenti, 1981).

In the “chromadoroid” three-part pharynx sensu Maggenti (1981), the corpus is sometimes subdivided into a pro- and metacorpus, with the latter showing only a minor swelling as in Plectida (e.g. Ohridiidae and Leptolaimidae) (Holovachov, 2014; Maggenti, 1981). The radial arms of the pharynx lumen often terminate as well-developed pharyngeal tubes in the corpus. When corpus and isthmus cannot be discerned by shape alone, a distinct change in tissue (Maggenti, 1981; Holovachov, 2004) as well as the position of the orifice of the subventral gland ducts (Holovachov, 2004), which are further indicated by”well visible cell borders” (Fürst von Lieven, 2003), can act as markers. In contrast to the corpus and posterior bulb, the isthmus lacks nuclei (Maggenti, 1981). Allen (1960) also mentioned the absence of nuclei inside the isthmus in three-part pharynges but without specifying them as a “chromadoroid” type. The bulbus is often equipped with a grinder.

However, this definition sensu Maggenti (1981) applies rather to the pharynx properties found in Plectida rather than those in the early branching Chromadorida, which usually have a two-part pharynx (Chitwood & Chitwood, 1977; Fürst von Lieven, 2003; Tchesunov, 2014a) with nuclei distributed along its entire length (Chitwood & Chitwood, 1977).

In Plectida, the isthmus lacks nuclei, and even in the genera *Anonchus* and *Aphanolaimus*, which both have a one-part pharynx, a distinct gap in the nuclei distribution is evident (Chitwood & Chitwood, 1977) corresponding to the position of the narrow isthmus in the genus *Plectus* and therefore to a potentially plesiomorphic isthmus prerequisite (Maggenti, 1981). A similar condition is also found in the genus *Terschellingia* (Monhysterida, Siphonolaimoidea), which shows a noticeable gap in its nuclei distribution in the middle of the two-part pharynx (Maggenti, 1963; Chitwood & Chitwood, 1977). In contrast, the nuclei maps of *Ethmolaimus* and *Chromadora* (both Chromadorida) show a regular nuclei distribution throughout the pharynx (Chitwood & Chitwood, 1977). Pharyngeal tubes, which are part of Maggenti’s definition of the “chromadoroid” three-part pharynx, have been reported for Plectoidea (Holovachov, 2004) but not for Chromadorida. Lastly, the lumen of the pharyngo-intestinal valve is dorso-ventrally flattened in Plectida but triradiate in Chromadorida (Chitwood & Chitwood, 1977). We therefore propose the term “plectoid” three-part pharynx instead of Maggenti’s “chromadoroid” three-part pharynx and will use it in the following text. At the same time, we propose to retain the name “chromadoroid” three-part pharynx for those three-part pharynges with no division of the corpus into pro- and metacorpus, a regular distribution of nuclei throughout the pharynx including the isthmus and a lack of pharyngeal tubes and grinders. Instead of a grinder, the posterior bulbus is often equipped with a crescentic valve (Tchesunov, 2014a, b). This type of pharynx is found within both Chromadorida and Desmodorida.

In Chromadorida, three-part pharynges with a noticeable anterior muscular swelling are present in subfamilies such as Hypodontolaiminae and Harpagonchinae (both Chromadoridae) and families such as Ethmolaimidae and Selachinematidae (both Chromadoroidea) in conjunction with mandibles (Selachinematidae and Harpagonchinae) or a large hollow dorsal tooth (Hypodontolaiminae) (Tchesunov, 2014a). In Ethmolaimidae, the anterior pharynx is either slightly widened enclosing the buccal cavity, or the buccal cavity is set off from the pharynx Filipjév & Stekhoven, (1941) and therefore does not build a three-part pharynx in a strict sense.

In the Desmodorida De Coninck (1965), there are only a few cases of a swollen corpus. Among the paraphyletic Microlaimoidea, *Bolbolaimus* and *Pseudomicrolaimus* (Microlaimidae) and *Synonema* (Aponchiidae) have an anterior pharyngeal swelling. In *Sigmophoranema* (Desmodoridae, Spiriniinae), the buccal cavity is equipped with an “S”-shaped dorsal tooth and several denticles and is surrounded by a pharyngeal swelling. Within Draconematidae (Desmodoroidea, Draconematidae), the corpus may be swollen. The prominently enlarged corpus (dumbbell-shaped pharynx) in *Dracognomus* was considered apomorphic within Draconematidae (Decraemer et al., 1997). With the notable exception of the Stilbonematinae (see below), these are the only reported cases of three-part pharynges within the Desmodorida (Tchesunov, 2014b).

There are a few more reports of three-part pharynges in Chromadoria that cannot be assigned to the above typology. In the Siphonolaimoidea (Monhysterida), a slightly widened corpus has been reported for *Sphaerocephalum* (Linhomoeidae, Linhomoeinae), *Sarsonia* (Linhomoeidae, Desmolaiminae) (Fonseca & Bezerra, 2014), and in *Siphonolaimus* (Siphonolaimidae, Siphonolaiminae); the corpus-isthmus transition is markedly constricted by the peripharyngeal nerve ring (Ott, 1972). The genera *Cyartonema* and *Paraterschellingia* (Cyartonematidae) possess a swollen corpus that is distinctly set off from the narrow isthmus (Tchesunov, 1989).

All above-mentioned cases of tripartite pharynges within Chromadorida, Desmodorida and Monhysterida are exceptional for these orders. They appear to reflect function rather than homology and therefore probably evolved several times independently. In none of these cases is the subdivision into corpus and isthmus considered plesiomorphic.

### The three-part pharynx found in Stilbonematinae

The three-part pharynx of Stilbonematinae can be distinguished from all three-part pharynx types mentioned by Maggenti by the following morphological aspects: (1) In the genera *Catanema*, *Robbea* and *Cyathorobbea*, a prominently swollen corpus is present and not subdivided into pro- and metacorpus (Gerlach, 1956; Gerlach, 1963; Platt & Zhang, 1982; Ott et al., 2020; Scharhauser et al., 2024; this study). (2) Both the buccal cavity and corpus are devoid of any armature. (3) The isthmus has regularly distributed nuclei throughout its length. (4) Neither the corpus nor the posterior bulb is valved. (5) No pharyngeal tubes are present. These differences (listed in Table 2) warrant the definition of a new pharynx type, the “stilbonematoid” three-part pharynx.

**Table 2:** Comparative table showing morphological differences of three-part pharynx types in Chromadoria. (“+” or “−” in parentheses indicate a rare condition — for details, see Holovachov, 2004)

Pharynx type	Buccal armature	Corpus subdivided	Nuclei in the isthmus	Pharyngeal tubes			Valve		
				Procorpus	Metacorpus	Isthmus	Procorpus	Metacorpus	Bulbus
Chromadoroid	Teeth/mandibles	−	+	−		−	−		+/−
Stilbonematoid	-	−	+	−		−	−		−
Plectoid	-	(+)/−	−	+	−	(+)/−	−	−	+/(−)
Rhabditoid	-	+	−	+	+/−	+	−	−	+
Diplogasteroid	Teeth	+	−	−	−	−	−	+	−
Tylenchoid	Spear	+	−	−	−	−	−	+	−

### Definition of the three-part pharynx found in Stilbonematinae

The three-part pharynx of Stilbonematinae (Fig. 3) consists of a corpus that is not subdivided into pro- and metacorpus, a well-developed intermediate isthmus and a spherical to ovoid posterior bulbus. The corpus is either sharply set off from the narrow isthmus in the genera *Catanema*, *Robbea* and *Cyathorobbea* or the corpus-isthmus transition is gradual, as is the case in *Laxus oneistus*. The pharynx is devoid of pharyngeal tubes and lacks valves either in the corpus or in the posterior bulb. Nuclei are distributed throughout the pharynx. The position of the gland ampullae is variable. Nonetheless, the typical chromadorean condition of the relative positions of dorsal and subventral gland ampullae to each other — with the dorsal ampulla at the anterior end of the pharynx and the subventral ampullae posterior to it (Chitwood & Chitwood, 1977) — is also present in Stilbonematinae. The one exception is *Leptonemella juliae* Hoschitz et al., 1999, in which the subventral ampullae are slightly anterior to the dorsal ampulla at the anterior end of the pharynx (Hoschitz et al., 2001). This is similar to the condition found in some Spiruromorpha (Chitwood & Chitwood, 1977).

### Myofilament density and nuclei

In genera with a cylindrical anterior pharynx, the posterior bulb is equipped with prominent musculature that is only slightly displaced by gland tissue (Fig. 1a, b and own unpublished data). We conclude that in these species, the bulbus is the main player in pumping activity. In species with a swollen corpus that contains most of the pharynx musculature, however, this function is assumed by this pharynx region. The conspicuous lobulation of nuclei inside the corpus is probably a spatial compromise with the density of musculature inside this pharynx part. The same accounts for the elongated nuclei in the slender isthmus. Similar nuclei deformations have, to our knowledge, so far only been reported for the pharyngeal myoepithelium of paucitubulatine gastrotrichs (Bekkouche & Worsaae, 2016) and for the epidermis of vestimentiferan metatrochophores (Bright et al., 2013).

### The value of the position of gland ampullae as a morphological trait

The relative position of gland ampullae is stable within individual genera even though the size of the corpus may differ greatly between species of the respective genus. An example is the genus *Cyathorobbea*, where the corpus of *Cy*. *agricola* is small compared to that of *Cy*. *ruetzleri*, but the relative position of the gland ampullae is identical. This positional stability is also present in the genera *Robbea* and *Catanema*. The position of the gland ampullae in the pharynx — one of the most complex morphological structures in nematodes — therefore appears to be valuable for phylogenetic interpretations. It can be used as a morphological marker to assess whether different amounts of the ancestral stilbonematoid pharynx were used to build the corpus in the genera *Catanema*, *Robbea*, *Cyathorobbea* and *Laxus*.

### The origin of the three-part pharynx in Stilbonematinae (morphological evidence and molecular support)

The pharynx in stilbonematine shows a surprising morphological diversity (Figs. 1 and 3).

The cylindrical condition of the anterior pharynx part of several Stilbonematinae genera such as *Eubostrichus*, *Leptonemella*, *Stilbonema*, *Eubostrichopsis* and species such as *Laxus cosmopolitus* Ott et al., 1995 and *L. sakihariiae* Leduc & Sinniger, 2018 is very similar to the two-part pharynges in closely related groups such as Desmodorinae and Spiriniinae (Tchesunov, 2014b). A cylindrical anterior pharynx part should therefore be regarded as plesiomorphic and a pronounced corpus swelling apomorphic. The hypothetical ancestral stilbonematoid pharynx most probably was a two-part pharynx without a buccal armature, without grinders or otherwise heavily cuticularized plates, without pharyngeal tubes, a cylindrical anterior part and a spherical posterior bulbus. Which parts of the ancestral pharynx have been incorporated into a swollen corpus in different Stilbonematinae genera may be assessed by the position of gland ampullae. In *Cyathorobbea* the gland ampullae are positioned at the anterior-most end of the isthmus. In both *Robbea* and *Catanema*, they are located more posterior, leaving enough space for nuclei in the anterior part of the isthmus. We conclude that a larger amount of the hypothetical ancestral Stilbonematinae pharynx was used to build the corpus in *Cyathorobbea* than in both *Robbea* and *Catanema.* The corpus swellings of the genera *Robbea* and *Catanema* should be considered homologous, while that of *Cyathorobbea* evidently evolved independently. This is supported by molecular data showing a closer relationship between *Robbea* and *Catanema* and a more distant phylogenetic relationship with *Cyathorobbea* (Scharhauser et al., 2024). *Laxus oneistus* is a special case: the subventral ampullae are in the centre of the corpus, which lacks a dorsal ampulla. This suggests that, compared to *Cyathorobbea*, an even larger amount of the ancestral pharynx was used to build the prominent corpus swelling in *Laxus oneistus*. This points to an origin independent of the muscular corpus of both *Cyathorobbea* and *Catanema* and *Robbea*, respectively.

### Functional assessment

Assessing the functional differences between those stilbonematoid pharynges with a more or less cylindrical corpus and those with a prominently swollen corpus is not a trivial task. Since stilbonematids do not feed after being extracted from their natural habitat (Ott et al., 1991), no data exist for a comparison with feeding observations in other nematodes (Doncaster, 1962, 1966; Fürst von Lieven, 2003; Mapes, 1965). The presence of a prominently muscular anterior corpus implies that the corpus itself is the main pumping structure in such a pharynx, similar to the prominent metacorpus within Diplogasteromorpha (Maggenti, 1981). The presence of heavier corpus musculature in groups such as Siphonolaimidae and Selachinematidae is associated with a special buccal armature that requires strong musculature. The buccal cavity of Stilbonematinae, however, is minute, and both the buccal cavity and the pharynx are devoid of any kind of armature. Food uptake must therefore rely on pharyngeal sucking. Several attempts have been made to establish biomechanical models to describe the general functional aspects of the nematode pharynx (Bennet-Clark, 1976; Mapes, 1966; Roggen, 1973, 1979, 1982). Roggen (1979) discusses the functional differences between a cylindrical pharynx portion and a spherical bulb. He concluded that a spherical bulb can produce twice the injection — but only half the suction pressure of a cylindrical pharynx part of comparable length and lumen diameter. A cylindrical pharynx has the ability to create strong suction pressure and can ingest large quantities of food, as was shown in *Ascaris lumbricoides* Linnaeus, 1758, where the cylindrical pharynx would fill half of its lumen before the contents would reach the intestine (Mapes, 1966). The terminal bulb, with its ability to create more injection pressure compared to a cylindrical pharynx part, has the task of injecting ingested food into the intestine (Roggen, 1979). The position of a spherical bulb along the pharynx should not influence its physical abilities to create injection or suction pressure. We therefore assume that this is also applicable to a spherical bulbous anterior structure, such as a swollen corpus.

### Gourmand versus gourmet feeding

It is reasonable to assume that pharynx morphology is an adaptation to the nature of food and the mode of feeding. Since Stilbonematinae almost exclusively feed on their symbionts, the differences between the various symbiont coat arrangements should be reflected by the differences in pharynx morphology. It appears that it is the geometry of the coat — thickness, multiple layers versus thin monolayers — rather than the shape of individual symbiont cells that correlates with pharynx types. Being able to ingest large amounts of food with the high suction pressure created by a cylindrical anterior pharynx region is advantageous only when the bacterial coat is thick. In fact, those genera possessing such a pharynx type (*Stilbonema*, *Leptonemella*, *Paralaxus*, *Eubostrichus*) either have a voluminous multilayer or complex monolayer coat. In contrast, in genera with symbionts arranged as a thin monolayer, swollen corpora are present, as in *Catanema*, *Robbea* or *Cyathorobbea*. This allows selective feeding on small portions of bacteria sucked from the thin coat. In the genus *Laxus*, we find several stages of development of corpus differentiation: in *L*. *cosmopolitus* Ott et al., 1995, *L*. *parvum* Armenteros et al., 2014 and *L*. *sakihariiae* Leduc & Sinniger, 2018, only slight dilatation of the anterior pharynx portion is evident; in *L*. *longus* Cobb, 1894 and *L*. *cobbi* Inglis, 1967 (as *Catanema cobbi*), a distinct corpus swelling twice as wide as the isthmus and occupying 24% and 39% of the pharynx length, respectively; in *L*. *oneistus*, the corpus swelling reaches its most prominent state where it occupies almost 50% of the pharynx length. The independent evolution of a distinctly swollen corpus indicates that pharynx morphology is driven by the geometry of the bacterial coat rather than phylogeny. While those genera with the more ancestral cylindrical pharynx remain culinary gourmands, the advanced pharynx types with a swollen corpus feed parsimoniously in gourmet style.

## Data Availability

Data is available from the corresponding author upon reasonable request.
